# Early diagnosis of colorectal cancer via plasma proteomic analysis of CRC and advanced adenomatous polyp 

**Published:** 2019

**Authors:** Setareh Fayazfar, Hakimeh Zali, Afsaneh Arefi Oskouie, Hamid Asadzadeh Aghdaei, Mostafa Rezaei Tavirani, Ehsan Nazemalhosseini Mojarad

**Affiliations:** 1 *Faculty of Paramedical Sciences, Shahid Beheshti University of Medical Sciences, Tehran, Iran*; 2 *School of Advanced Technologies in Medicine, Shahid Beheshti University of Medical Sciences, Tehran, Iran*; 3 *Basic and Molecular Epidemiology of Gastroenterology Disorders Research Center, Research Institute for Gastroenterology and Liver Diseases, Shahid Beheshti University of Medical Sciences, Tehran, Iran*; 4 *Proteomics Research Center, Faculty of Paramedical Sciences, Shahid Beheshti University of Medical Sciences, Tehran, Iran*; 5 *Gastroenterology and Liver Diseases Research Center, Research Institute for Gastroenterology and Liver Diseases, Shahid Beheshti University of Medical Sciences, Tehran, Iran*

**Keywords:** Colorectal cancer, Advanced adenomatous polyp, Early detection, Plasma biomarker, proteomics

## Abstract

**Aim:**

This paper aimed to identify new candidate biomarkers in blood for early diagnosis of CRC.

**Background:**

Colorectal cancer (CRC) is the third most widespread malignancies increasing globally. The high mortality rate associated with colorectal cancer is due to the delayed diagnosis in an advanced stage while the metastasis has occurred. For better clinical management and subsequently to reduce mortality of CRC, early detection biomarkers are in high demand.

**Methods:**

A 2D-PAGE separation of proteins was performed followed by tandem mass Spectrometry (MALDI-TOF-TOF) to discover potential plasma protein markers for CRC and AA (advanced adenomas). Furthermore, western blot method was used to confirm a part of the results in colorectal tissue samples.

**Results:**

The significantly altered proteins including HPR, HP, ALB, KRT1, APOA1, FGB, IGJ and C4A were down-regulated in polyp relative to normal, and CRC compare to polyp surprisingly, and inversely, ORM2 was up-regulated with the fold change ≥ 2 and p-value ≤ 0.05. We also surveyed APOA1, FGB, and C4A for further confirmation of their expression changes by western blotting. All three of them showed a decreasing trend from normal toward CRC tissue samples as it mentioned before, but just changes of FGB and C4A were significant.

**Conclusion:**

The results demonstrated that plasma proteins can be less invasive markers for the detection of CRC. FGB and C4A can be considered as plasma potential biomarkers to early diagnosis of CRC patients and understanding the underlying procedures in tumorigenesis. Undoubtedly, the additional study must be conducted on large scale cohorts to verify the results.

## Introduction

 Overall, there were 14.1 million new cases of cancer and 8.2 million mortality in 2012. The most commonly diagnosed cancers were lung (1.82 million), breast (1.67 million), and colorectal (1.36 million) ones and the 3 most common causes of cancer mortality were cancers of the lung (1.6 million deaths), liver (745,000 deaths), and stomach (723,000 deaths). Based on the GLOBOCAN 2018, It was estimated that the number of new cases was nearly 18 million in 2018, worldwide, all cancers, both sexes, and all ages. Colorectal cancer will be the third most common cancer (1.8 million) after lung (2.09), and breast (2.08) ones. The best available data on cancer incidence and mortality in the form of tabulation and graphical visualization of the full dataset released by 184 countries and 30 world regions by sex can be accessed via the GLOBOCAN homepage (http://globocan.iarc.fr) (1). Morbidity and mortality arising from cancer are rapidly growing worldwide. There are several complex reasons, but the main one is the change in the spread of the cancer risk factors associated with socioeconomic development in modern societies (2). 

With colorectal cancer (CRC) patients, the high mortality rate is mainly due to the delayed diagnosis of cancer in its advanced stage, while the metastasis has occurred. The 5-year survival rate of CRC patients diagnosed at the early, and late stage of cancer was reported nearly 90% and only less than 10% respectively (3, 4). The pathogenic mechanisms related to CRC development are complex and heterogeneous (5).

About two-thirds of all CRCs, sporadic colorectal cancer, develops from the benign tumor of glandular epithelial tissue without a recognized family history or germline causes of cancer or inflammatory bowel disease. These precursors named adenomatous polyp, which is initially noninvasive but progresses slowly into cancer within 10 to 20 years in approximately 5% of patients. As a screening test for colorectal cancer, we should consider the advanced adenomas as an adenoma with the size of 10 mm or more, a villous adenoma, or high-grade dysplasia that is most likely to progress into carcinoma. It is probably an excellent opportunity to detect and remove premalignant polyps and other precancerous lesions to prevent cancer incidence (6-10). 

Evidence exists that early detection of CRC is the most important key point to reduce the associated mortality rate. Unfortunately, the lack of overt clinical symptoms in the primary stages makes it difficult to find and treat CRC patients effectively. The invasive, unpleasant, low-compliance, insignificant sensitivity and accuracy of the current diagnostic methods for screening individuals which include a fecal occult blood test (FOBT), colonoscopy and carcinoembryonic antigen (CEA) blood test demands an urgent need to substitute them with a non-invasive approach like the blood-based test. Despite the importance of early detection, some people throughout the world have never been screened for CRC. Hence, it is essential to seek for novel biomarkers with high sensitivity and specificity to diagnose and treat CRC successfully and in due course of time, thereby increasing the survival rate of patients (11-13).

Proteomics is an efficient and beneficial approach for hunting candidate cancer biomarkers, as we can analyze multiple proteins with different expressions in one study simultaneously (4). Two-dimensional electrophoresis (2-DE) and mass spectrometry (MS) are suitable and promising proteomic techniques with an adequate resolving power of proteins mixture upon a unique platform simultaneously. Furthermore, it can detect peptide fragments with post-translational modification and amino acid mutation; however, 2-DE suﬀers from some limitations in hydrophobicity, size, and solubility of protein samples. Nevertheless, researchers have identified different novel proteins/peptides through utility of 2-DE and MS analysis (3, 14, 15) in Breast (16-19), Brain (20-22), Lung (23-27), Colon (28-30), Renal (31-34), Ovarian (35-39) cancer and Bone marrow (40).

There are no adequately sensitive and specific blood-based biomarkers for early diagnosis, although, a convenient way to estimate the internal state of the human body is blood (41-43). In this report, we aim to identify plasma proteins that can discriminate between healthy control, advanced adenoma and CRC patients in early stages and therefore have the potential to function as biomarkers for non-invasive early detection. We used 2-DE/MS to investigate expression changes of plasma proteins related to adenomatous polyp subjects and CRC patients with an incremental or decreasing gradient than healthy subjects. 

## Methods

**Patient/sample information**

In this cohort study, multiple groups of human plasma samples including 30 normal, 30 AA (advanced adenomas), and 30 grade 1 or 2 of CRC (colorectal cancer) patients who had not been undergone any clinical treatment before the time of sample acquisition were analyzed and compared in terms of protein expression. All the patient samples were obtained from the Taleghani Hospital, Tehran, Iran. The clinical ethics committee approved this study of the Shahid Beheshti University of Medical Sciences. All the participants provided written, informed consent to enter the study. Subjects had a median age of 47 years (ranging from 24 to 66) including 47 females and 43 males and were the age- and gender-matched with each other in three groups. Blood samples of suspicious people were collected into EDTA tubes and processed identically in Gastroenterology and Liver Disease Research Center, Shahid Beheshti University of Medical Sciences, Tehran, Iran. As well as the biopsy samples were taken from inside of the bowel by a loop and were sent to detection laboratory. Diagnosis of all the AAs and CRCs were confirmed pathologically in the pathology laboratory of Taleghani Hospital. 

According to inclusion criteria, the control subjects in this project are people who do not have first-degree family members with any cancer, and it will be determined that they are healthy and have no polyps after colonoscopy. To confirm the lack of inflammation in healthy people, C-reactive protein (CRP) test was done. Positive people were excluded from the study. By the survey of the pathologic outcomes of patients, in the second group, subjects who had no advanced-stage polyps were excluded from the research and among the cancer patients group, patients in stages 3 and 4 were excluded from the study.

The blood samples were transported to the laboratory for 10 minutes and centrifuged immediately two times at 3700 rpm for 10 min at 4˚C. Pipette the plasma into a clean plastic vial and attach the label on it. Finally, the plasma samples were stored at -80˚C for further processes. 

**Two-dimensional gel electrophoresis (2-DE)**

All 2-DE chemicals and Ready Strip™ IPG strips in this stage were obtained from SERVA Company, GE HealthCare Life Sciences, and Sigma Company. Protein extraction was carried out by the acetone precipitation method in accordance with the introduction of Sigma ProteoPrep Protein Precipitation Kit. First, the protein concentration of all samples in three groups was measured by Bradford assay. The 2-DE procedure was done with three times replications of normal, AA and CRC samples. The 1st dimension of electrophoresis, isoelectric focusing (IEF), was conducted by use of Bio-Rad Protein IEF Cell, 11cm nonlinear IPG with the pH range of 3 to 11 for 7.5 h at 20°C according to Bio-Rad protocol. The strips have to be rehydrated before used for 8 hours. In following sections, 1400μg protein was loaded to the tray per gel in the IEF phase. In this stage, proteins were separated based on their isoelectric point (pI). Prior to the second dimension, you need to equilibrate the IPG strips for 30 minutes at room temperature. Subsequently, proteins were separated based on Molecular Weight (MW) in the second dimension that consists of 12% sodium dodecyl sulfate-polyacrylamide gel electrophoresis (SDS-PAGE). After the segregation of proteins, the gels were dyed by Coomassie blue stating method (3L including 0.3 g Coomassie R250, 18 mL orthophosphoric acid, 90 mL acetic acid). Then, they were scanned into a computer using a Bio-Rad scanner. In the subsequent stage, the protein expression profiles were analyzed by employing Progenesis SameSpots software (Bio-Rad). Finally, the significant differentially expressed proteins having a gradual upward or downward expressed change from the normal mode toward the polyps and cancer mode, respectively, were selected and cut upon gels. 

**In-gel Trypsin Digestion**

Gel pieces were washed two times with 50% (v:v) aqueous acetonitrile containing 25 mM ammonium bicarbonate, then once with acetonitrile and dried in a vacuum concentrator for 20 min. Sequencing-grade, modified porcine trypsin (Promega) was dissolved in the aqueous 50 mM acetic acid, then diluted 5-fold with 25 mM ammonium bicarbonate to give a final trypsin concentration of 0.02 µg/µL. Gel pieces were rehydrated with 10 µL of trypsin solution, and after 10 min, an adequate 25 mM ammonium bicarbonate solution was added on to cover the gel pieces. Digests were incubated overnight at 37^o^C.

**MALDI-MS/MS**

Matrix-associated laser desorption ionization-time of flight mass spectrometry (MALDI-TOF/MS) is one of the most powerful tools. The differentially expressed peptides, proteins are ionized and separated based on the mass-to-charge ratio (*m/z*) using a mass spectrometer (44). A 1µL aliquot of each peptide mixture was applied to a ground steel MALDI target plate, followed immediately by an equal volume of a freshly-prepared 5 mg/mL solution of 4-hydroxy-a-cyano-cinnamic acid (Sigma) in 50% aqueous (v:v) acetonitrile containing 0.1 %, trifluoroacetic acid (v:v). Positive-ion MALDI mass spectra were obtained using a Bruker ultraflex III in reflectron mode, equipped with an Nd:YAG smart beam laser. MS spectra were acquired over a range of 800-4000 *m/z*. Final mass spectra were externally calibrated against an adjacent spot containing 6 peptides (des-Arg-Bradykinin, 904.681; Angiotensin I, 1296.685; Glu-Fibrinopeptide B, 1750.677; ACTH (1-17 clip), 2093.086; ACTH (18-39 clip), 2465.198; ACTH (7-38 clip), 3657.929.). Monoisotopic masses were obtained using a SNAP averagine algorithm (C 4.9384, N 1.3577, O 1.4773, S 0.0417, and H 7.7583) and an S/N threshold of 2. Ten strongest precursors per spot, with an S/N greater than 30, were selected for MS/MS fragmentation. Fragmentation was performed in LIFT mode without the introduction of a collision gas. The default calibration was used for MS/MS spectra, which were baseline-subtracted and smoothed (Savitsky-Golay, width 0.15 *m/z*, cycles 4); monoisotopic peak detection used a SNAP averagine algorithm (C 4.9384, N 1.3577, O 1.4773, S 0.0417, H 7.7583) with a minimum S/N of 6. Bruker flexAnalysis software (version 3.3) was used to perform spectral processing and peak list generation. Tandem mass spectral data were submitted to database searching against the human subset of the SwissProt database (20259 sequences; 11273696 residues) using a locally-running copy of the Mascot program (Matrix Science Ltd., version 2.5.1), through the Bruker ProteinScape interface (version 2.1). Search criteria specified: Enzyme, Trypsin; Fixed modifications, Carbamidomethyl (C); Variable modifications, Oxidation (M) and Deamidated (NQ); Peptide tolerance, 50 ppm; MS/MS tolerance, 0.5 Da; Instrument, MALDI-TOF-TOF Ultraflex instrument. Results were filtered to accept only peptides with an expected score of 0.05 or lower.

**Verification by Western blots**

Tissues were harvested and lysed in lysis buffer (50 mM Tris-HCl, 150 mM NaCl, 0.1% SDS, 100ug/ml PMSF, 1ug/ml Aprorinin, 1% NP-40) in the presence of protease inhibitors. Lysates were mixed well and put into ice between 30 and 60 min. Then they were centrifuged 10 min at 10000 rpm and 4^o^, after that, the supernatant was taken into a new tube. The Lowry assay then defined protein concentration. The samples were added to the same amount of 2X loading buffer. After incubation at 95^o^ for 5 min, each sample was loaded into the bottom of wells placed on top of SDS-PAGE (the percentage of SDS-Polyacrylamide Gel was chosen according to molecular protein size). The electrophoresis apparatus was attached to an electric power supply and Run at 95v, 200A and in 190 min. Then, the gel was removed from the electrophoresis apparatus and incubate it in Western Transfer Buffer (10% methanol, 24 mM Tris, and 194 mM glycine) for approximately 10 min to remove detergent. Also, polyvinylidene ﬂuoride (PVDF) membrane was soaked into methanol for 5 min. And then, the samples were transferred onto PVDF membranes by making a blotting sandwich. The membranes were blocked with 5-10% (w/v) nonfat dried milk (Bio-Rad, Richmond) in PBS for 1 hour or overnight at 4^o^ with shaking. After that, they incubated with primary antibody against APOA1, FGB and C4A in 1% BSA in Tbst for 2 hours at room temperature. In the following, with a tandem repeat of washing, samples were incubated with secondary antibody (peroxidase-conjugated goat anti-mouse IgG, etc.) Depend on company data sheet; 1 hour at room temperature. DAB (3, 3’-diaminobenzidine) was poured on the membrane for monitoring and visualizing the bands.

**Statistical Analysis**

The protein expression profiles were analyzed by employing Progenesis SameSpots software. Predetermined criteria were the value of 2≤fold increase or decrease and P-value≤0.05 in protein spots which were identified using 1-way ANOVA analysis. Furthermore, we used DAVID Bioinformatics Resources 6.8 (https://david.ncifcrf.gov/) (45) and GeneCards – the human gene database (www.genecards.org) (46) aimed to retrieve annotation summary results like Kegg Pathway and Gene Ontology related to intended proteins. 

## Results

**Figure 1:**
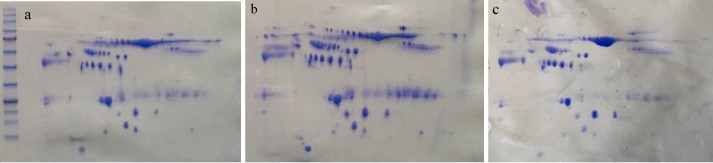
a) The protein profile of control samples. b) The protein profile of advanced adenomas samples. c) The protein profile of colorectal cancer samples separated by two-dimensional gel electrophoresis (2-DE) method

Total protein extracts from normal, AA (advanced adenoma) and CRC (colorectal cancer) patients' plasma were separated by 2-DE (figure 1). Analysis after Coomassie blue staining was performed by Progenesis SameSpots software, followed by MALDI‐MS and SWISS-PROT database. Acquired results have been tabulated in table 1. Results showed that the expression levels of HPR (haptoglobin-related protein), HP (haptoglobin), ALB (Serum albumin), KRT1 (Keratin, type II cytoskeletal 1), APOA1 (Apolipoprotein A-1), FGB (Fibrinogen beta chain), IGJ (Immunoglobulin J chain) and C4A (Complement C4-A) were lower in advanced polyp sample compared to normal one and as well as, they were lower in cancer sample rather than polyp one.

**Table 1:** The list of identified nine protein spots with some information inferred from MASCOT (P<0.05)

Protein Name	UniProt Code	Protein Seq Coverage (%)	Peptide Matches	Matching Score	Advanced polyp vs. normal	Early CRC vs. advanced polyp
Serum albumin	P02768	10	5	392	down	down
Haptoglobin	P00738	14	6	352	down	down
Haptoglobin-related protein	P00739	6	4	305	down	down
Keratin, type II cytoskeletal 1	P04264	11	5	241	down	down
Immunoglobulin J chain	P01591	28	4	309	down	down
Apolipoprotein A-I	P02647	27	7	475	down	down
Fibrinogen beta chain	P02675	7	3	181	down	down
Complement C4-A	P0C0L4	1	4	209	down	down
Alpha-1-acid glycoprotein 2	P19652	21	4	278	up	up

**Figure 2:**
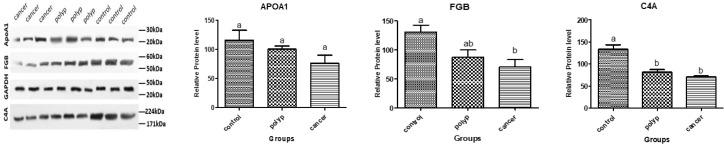
Comparison of relative protein level of APOA1, FGB and C4A between control, polyp and cancer tissues taken from western blotting test. GAPDH is control

On the contrary, the expression level of ORM2 (Alpha-1-acid glycoprotein 2) was higher in advanced polyp and cancer than a polyp. Nine significantly altered proteins with their properties were tabulated in table 2. We searched the protein names in GeneCards, which is a searchable, integrative database that provides comprehensive, user-friendly information on all annotated and predicted human genes. It automatically integrates gene-centric data from ~150 web sources including genomic, transcriptomic, proteomic, genetic, clinical and functional information. Moreover, we surveyed the role of these genes in various cancers via search engines and a study published in the literature.

Three proteins, including APOA1 (Apolipoprotein A-1), FGB (Fibrinogen beta chain) and C4A (Complement C4-A) were considered for further evaluation by western blotting. The reason for our choice was that FGB and C4A had not been identified before as a candidate biomarker for the diagnosis of CRC. Whereas, ORM2 was a good factor for further confirmation of expression changes, because it was up-regulated in CRC samples and can be an excellent diagnostic biomarker, but since it has already been validated with Enzyme-Linked Immunosorbent Assay (ELISA) and Western blot in other studies (78, 79), we gave up to reconfirm this protein. Concerning many studies performed on APOA1 and its expression pattern changes in many cancers, we verified APOA1 downregulation by western blot as a control for our project. 

**Table 2:** Some characteristics of the identified differentially expressed proteins of plasma samples from patients with CRC and AA compared to control group published in Genecards database and literatures

Protein name	Information from Genecards	Information retrieved from Published literatures
HPR (haptoglobin-related protein)	Clinically important predictor of recurrence of breast cancer.	Immunohistochemical staining confirmed the absence of haptoglobin in normal colon and the ectopic expression of haptoglobin in colon cancers and adenomatous polyps (47). HPR in laryngocarcinoma patients were downregulated (48). HPR -epitope expression is a clinically important predictor of the recurrence of cancer in patients with early breast cancer, especially in combination with progesterone-receptor status (43).
HP (haptoglobin)	Plays a role in intestinal permeability.	Higher haptoglobin levels were linked with an increased risk of colorectal cancer death (49). Serum HP is associated with poor prognosis of CRC patients and that HP promotes colorectal cancer cell invasion (50). Combination of haptoglobin and osteopontin could predict colorectal cancer hepatic metastasis (51). Distinctive expression pattern of HP in breast, colorectal and lung cancer (52).
ALB (Serum albumin)	Regulation of the colloidal osmotic pressure of blood. Major zinc transporter in plasma. Carrier protein for water, Ca (2+), Na (+), K (+), fatty acids, hormones, bilirubin and drugs. Putative down-regulated c-Myc target gene.	Metastatic colon cancer show relatively small decline of ALB compared to the mean in tumor-free patients (53). The preoperative CRP/ALB ratio is a useful prognostic marker in patients with colorectal and ovarian cancer who undergo potentially curative surgery (54-57). Alb is an important prognostic factor for patients with Hepatocellular carcinoma (HCC) (58).
KRT1 (Keratin, type II cytoskeletal 1)	Binding protein and presentation for kininogens on endothelial cells. May regulate the activity of kinases.	KRT1 as a new marker for breast cancer targeting that is highly expressed on breast cancer cells (59). Upregulation of Keratin inhibits the invasion of MDA-MB-231 breast cancer cells (60). Keratin 1 has higher expression in nasopharyngeal carcinoma cell lines (61).
APOA1 (Apolipoprotein A-1)	The major protein of HDL, involved in the reverse transport of cholesterol from tissues to liver for excretion.	Patients with CRC exhibited significantly increased APOA1 levels after adjuvant chemotherapy (62). The higher amounts of APOA1 reduce breast, colorectal, and lung cancer risk (63). ApoA1 is inversely associated with lung cancer risk (64). ApoA1 is closely related to breast cancer (65). ApoA1 as a biomarker for diagnosis of Pancreatic carcinoma (PC) (66).
FGB (Fibrinogen beta chain)	Various cleavage products of fibrinogen and fibrin regulate cell adhesion and spreading. Are mitogens. Signaling by moderate kinase activity BRAF mutants. Fibrinogen is cleaved by thrombin to form fibrin which is the most abundant component of blood clots.	FGB has prognostic value for lung adenocarcinoma (67). FGB in combination with other four urinary biomarkers not only discriminates lung cancer patients from control groups but also differentiates lung cancer from other common tumors (68). Elevated plasma fibrinogen levels and tumor progression in patients with gastric cancer have been largely reported (69). Increased expression of FGB was correlated with increased bladder tumor stage and can be a potential diagnostic marker (70).
IGJ (Immunoglobulin J chain)	Joining Chain Of Multimeric IgA And IgM. Among its related pathways are Cell surface interactions at the vascular wall.	The abundance of immunoglobulin J chain (IGJ) is significantly higher in CRC mice than in control mice (71). IGJ can predict outcome and relapse in paediatric pre-B acute lymphoblastic leukaemia (72). The expression level of IGJ was downregulated in esophageal cancer (73). IGJ was downregulated during breast cancer progression in tumor-associated stroma (74).
C4A (Complement C4-A)	Among its related pathways are Sudden Infant Death Syndrome (SIDS) Susceptibility Pathways.	C4A can be used for predicting metachronous liver metastasis (MLM) in patients who underwent radical resection of colorectal cancer (75). C4A is a potential biomarker for the diagnosis of Hepatocellular carcinoma (HCC) (76). C4A is less expressed in plasma of patients suffering from squamous cell carcinoma of the penis (SCCP) in comparison with healthy subjects (77).
ORM2 (Alpha-1-acid glycoprotein 2)	A key acute phase plasma protein. Among its related pathways are Innate Immune System and Response to elevated platelet cytosolic Ca2+. It may be involved in aspects of immunosuppression. As transport protein in the blood stream binds synthetic drugs and influences their distribution and availability.	Plasma ORM2 is an independent prognostic factor both for overall and for cancer-specific survival in patients with stage II CRC (78). ORM2 could be used as a potential biomarker in the diagnosis of CRC (79). Overexpression of Plasma ORM2 could serve as a new risk marker for Cholangiocarcinoma (CCA) (80-82). ORM1 and ORM2 plasma concentrations are increased in breast, lung, and ovary cancers (83).

All three proteins showed a decreasing trend in tissue samples of the mentioned three groups (Fig. 2). 

## Discussion

Such a proteomic analysis of 30 colorectal cancer, 30 advanced adenomas, and 30 normal plasma samples provides several insights into the biology of CRC and identifies potential plasma biomarkers. Since our goal is to identify the proteins that promote the progression of healthy tissue to advanced adenomas and then to cancer, thus, among all differentially expressed proteins we chose proteins that had a continuous change in three groups respectively. We identified eight proteins (HPR, HP, ALB, KRT1, APOA1, FGB, IGJ, and C4A) which are down-regulated in the polyp relative to the normal. Surprisingly, these proteins are down-regulated in the CRC compared with the polyp. The ninth introduced protein is ORM2 that is up-regulated in the polyp and cancer relative to the normal and polyp, respectively. 

After antibody validation by Western blotting, it was verified that APOA1 (Apolipoprotein A-1) is down-regulated in two processes; normal toward polyp and subsequently cancer. Down-regulation of APOA1 in CRC has been reported previously (62, 63). This finding confirms our results because APOA1 is one of identified nine proteins in our research. FGB (Fibrinogen beta chain) represents significant down-regulation changes in both processes; transformation of normal into polyp tissue and polyp into cancerous tissue, however, C4A expression decreased significantly solely in polyp rather than normal tissue (figure 2). 

Fibrinogen is an acute‐phase coagulation factor which is predominantly produced by hepatocytes and activated by thrombin to be converted to fibrin. It is covalently cross-linked by coagulation factor XIIIa participated in the final stages of blood clotting to form fibrin fibers, which are the main components of blood clots (84, 85). Systemic activation of the coagulation mechanism has been reported in patients with colon cancer (86). However, it should be noted that abnormalities of coagulation and fibrinolysis play an important role in cancer progression (87, 88). Fibrinogen inside tumor cells plays a crucial role in the pathophysiology of tumor cell growth and metastasis (89, 90). Fibrinogen, fibrin, and their degradation products possess a proinflammatory role in the pathogenesis like vascular wall disease, brain trauma, Alzheimer's disease, rheumatoid arthritis, bacterial infection, colitis, lung and kidney fibrosis, Duchenne muscular dystrophy, and several types of cancer may lead to complications of thromboembolic events. They can stimulate endothelium indirectly to secrete von Willebrand factor, leading to the activation of platelets. 

Fragments X, Y, E, and D are the end products of fibrinogen degradation, and D-dimer, which consists of two D fragments cross-linked by a γ- chain is the end product of stabilized fibrin as a result of plasmin cleavage during fibrinolysis. Fragment E stimulates proliferation, migration, and differentiation of endothelial cells, contributing to tumor vasculature. Increased levels of DD are observed in malignant neoplasms such as breast, lung, colon, and ovary cancers. The high level of D-dimer is found in patients with disseminated intravascular coagulation, thromboembolic complications, during increased fibrinolysis. The activation of both clotting and fibrinolytic systems has been found in breast cancer. CG Mitter et al. declared plasma levels of D-dimer and thrombin-antithrombin-III complex (TAT), reflecting the activation of thrombin, are significantly increased in patients with breast cancer, as compared to healthy controls.

Furthermore, CG Mitter showed significant correlations between plasma concentrations of D-dimer and serum levels of carcinoembryonic antigen (CEA) and CA15-3, respectively (91-93). Some regulatory factors of ﬁbrin activation/degradation expressed on cancer cell surfaces may also be important in tumor invasion, proliferation, and metastasis (94). Recent studies have highlighted important parallels between wound healing and cancer at the molecular and cellular level. The wound-healing process occurs in three overlapping phases: inflammation, new tissue formation, and tissue remodeling (95).

C4A is complement protein encoded in the class III region of major histocompatibility complex (MHC). Its expression regulation is under genetic and hormonal control (96). Complement is a central part of the innate immune response, which is the first defense against microbes and undesirable host elements in a non-speciﬁc manner, as well as, it arranges many processes contribute to homeostasis. C4 complement fragment is involved in the classical complement cascade pathway. The activated C1 removes a small fragment of C4 alpha chain named C4A anaphylatoxin. The big remaining alpha chain fragment C4B is the major activation product and is an essential subunit of the C3 convertase (C4B2A) and the C5 convertase (C3BC4B2A) enzymes of the classical complement pathway. C4A anaphylatoxin is a mediator of the local inflammatory process (45). Complement activation and generation of anaphylatoxins (C3A and C5A) in the tumor microenvironment increases tumor growth and metastasis. The anaphylatoxin receptors are G protein-coupled receptors presented on many cell types, including lymphocytes, monocytes/macrophages, myeloid cells, hematopoietic stem cells, mesenchymal cells, and epithelial cells which include cancer cells. (97). 

Complement interferes in various processes include adaptive immunity, humoral immunity, removal of apoptotic cells, regulation of the coagulation system, angiogenesis, hematopoietic stem/progenitor cell mobilization, regeneration of tissue, and lipid metabolism (98, 99). Recent studies have declared new roles for the complement system in the extravascular and interstitial tissue compartment. They illustrated those complement proteins can be important factors in cell-cell and stroma-cell communications (97). 

The anaphylatoxins are rapidly inactivated in plasma by carboxypeptidases, particularly carboxypeptidase N, and complement regulatory proteins (CRPs) perform their function on the cell surface. One hypothesis about cancer cells is that they can resist complement attack employing overexpressing CRPs. CRPs are overexpressed by many cancer cells and may be used as potential therapeutic targets (97). Degradation of complement system convertases was observed for patients with breast cancer (77).

Ornellas P et al. showed that C4A is downregulated in patients suffering from squamous cell carcinoma of the penis (SCCP). The results prove that it is more under-expressed when the disease progresses. Their hypothesis states that viral proteins from viruses highly prevalent in SCCP lesions counteract the immune response. This could explain the cause of downregulation of C3 and C4A/B along with the progression of the disease (77). Taneja et al. declared the plasma levels of complement proteins C3, C3f, C4, and bradykinin and kininogen had been decreased in the plasma of hepatitis E patients compared to healthy controls. The exact mechanism for this reduction was not understood at that time (100).

Pathways related to FGB and C4A were retrieved via the Kyoto Encyclopedia of Genes and Genomes (KEGG) through DAVID Bioinformatics Resources. Pathways associated with FGB include complement and coagulation cascades, Platelet activation. As well as, Pathways related to C4A include complement and coagulation cascades, Pertussis, Staphylococcus aureus infection, Systemic lupus erythematosus. Only one common pathway; complement and coagulation cascades (P-value= 1.4E-2) was determined. 

The most comprehensive study of the different aspects of the subject at a much broader level and with a large number of samples accompanying other high throughput techniques should be taken into serious consideration for getting more accurate and reliable results. Furthermore, we can gain better insight into the underlying processes by identifying more significant proteins and surveying them in cancerous pathways. 

Plasma proteins can be less costly and invasive markers for detection of CRC, So more people are willing to do this test. Consequently, the incidence of screening and early detection of CRC will increase while the related mortality will decrease. The results show that FGB and C4A may be involved in pathways which are caused by advanced adenomas and subsequently colorectal cancer. The pathway analysis as to such proteins reveals that the complement and coagulation cascades pathway plays an important role in the carcinogenesis. In conclusion, the data suggest that FGB and C4A can be considered as plasma potential biomarkers so that diagnosis of CRC patients and understanding the underlying procedures in tumorigenesis to be accelerated; although, it demands more extensive and cohorts studies. 

## References

[1] Ferlay J, Soerjomataram I, Dikshit R, Eser S, Mathers C, Rebelo M (2015). Cancer incidence and mortality worldwide: sources, methods and major patterns in GLOBOCAN 2012. Int J Cancer.

[2] Bray F, Ferlay J, Soerjomataram I, Siegel RL, Torre LA, Jemal A (2018). Global cancer statistics 2018: GLOBOCAN estimates of incidence and mortality worldwide for 36 cancers in 185 countries. Cancer J Clin.

[3] Lee PY, Chin SF, Low TY, Jamal R (2018). Probing the colorectal cancer proteome for biomarkers: Current status and perspectives. J Proteomics.

[4] Álvarez-Chaver P, Otero-Estévez O, de la Cadena MP, Rodríguez-Berrocal FJ, Martínez-Zorzano VS (2014). Proteomics for discovery of candidate colorectal cancer biomarkers. World J Gastroenterol.

[5] Arroyo R, Duran-Frigola M, Berenguer C, Soler-López M, Aloy P (2014). Charting the molecular links between driver and susceptibility genes in colorectal cancer. Biochem Biophys Res Commun.

[6] Wisniewski JR, Dus-Szachniewicz K, Ostasiewicz P, Ziolkowski P, Rakus D, Mann M (2015). Absolute Proteome Analysis of Colorectal Mucosa, Adenoma, and Cancer Reveals Drastic Changes in Fatty Acid Metabolism and Plasma Membrane Transporters. J Proteome Res.

[7] Winawer SJ, Fletcher RH, Miller L, Godlee F, Stolar M, Mulrow C (1997). Colorectal cancer screening: clinical guidelines and rationale. Gastroenterology.

[8] Wolf AM, Fontham ET, Church TR, Flowers CR, Guerra CE, LaMonte SJ (2018). Colorectal cancer screening for average‐risk adults: 2018 guideline update from the American Cancer Society. Cancer J Clin.

[9] Carethers JM, Jung BH (2015). Genetics and genetic biomarkers in sporadic colorectal cancer. Gastroenterology.

[10] Kim DH, Pickhardt PJ, Taylor AJ (2007). Characteristics of Advanced Adenomas Detected at CT Colonographic Screening: Implications for Appropriate Polyp Size Thresholds for Polypectomy Versus Surveillance. Am J Roentgenol.

[11] Zhai XH, Yu JK, Yang FQ, Zheng S (2012). Identification of a new protein biomarker for colorectal cancer diagnosis. Mol Med Rep.

[12] Dillon R, Croner LJ, Bucci J, Kairs SN, You J, Beasley S (2018). Analytical validation of a novel multiplex test for detection of advanced adenoma and colorectal cancer in symptomatic patients. J Pharm Biomed Anal.

[13] Ward D, Suggett N, Cheng Y, Wei W, Johnson H, Billingham L (2006). Identification of serum biomarkers for colon cancer by proteomic analysis. Br J Cancer.

[14] Doustjalali SR, Bhuiyan M, Al-Jashamy K, Linn NH, Abdul Kadir S, Appalanaidu V (2014). Two dimensional gel electrophoresis: An overview of proteomic technique in cancer research. Journal of Proteomics and Bioinformatics.

[15] Kuppusamy P, Govindan N, Yusoff MM, Ichwan SJA (2017). Proteins are potent biomarkers to detect colon cancer progression. Saudi J Biol Sci.

[16] Da Costa GG, Gomig THB, Kaviski R, Sousa KS, Kukolj C, De Lima RS (2015). Comparative proteomics of tumor and paired normal breast tissue highlights potential biomarkers in breast cancer. Cancer Genomics Proteomics.

[17] Gajbhiye A, Dabhi R, Taunk K, Vannuruswamy G, RoyChoudhury S, Adhav R (2016). Urinary proteome alterations in HER2 enriched breast cancer revealed by multipronged quantitative proteomics. Proteomics.

[18] Akpinar G, Simsek T, Guler A, Kasap M, Canturk NZ (2017). Elucidation of a conserved proteomic pattern of breast cancer tissue and metastatic axillary lymph node. Chirurgia.

[19] He J, Ma G, Qian J, Zhu Y, Liang M, Yao N (2017). Interaction between ezrin and cortactin in promoting epithelial to mesenchymal transition in breast cancer cells. Int Med J Exp Clin Res.

[20] Zhan X, Yang H, Peng F, Li J, Mu Y, Long Y (2018). How many proteins can be identified in a 2DE gel spot within an analysis of a complex human cancer tissue proteome?. Electrophoresis.

[21] Tsangaris GT, Dimas K, Malamou A, Katsafadou A, Papathanasiou C, Stravopodis DJ (2017). Molecular proteomic characterization of a pediatric medulloblastoma xenograft. Cancer Genomic Proteomic.

[22] Jain R, Atak A, Yeola A, Srivastava S (2017). Proteomic level changes associated with S3I201 treated U87 glioma cells. J proteomics.

[23] Peng B, Lei N, Chai Y, Chan EK, Zhang JY (2015). CIP2A regulates cancer metabolism and CREB phosphorylation in non-small cell lung cancer. Mol Biosyst.

[24] Amin A, Bukhari S, Mokhdomi TA, Anjum N, Wafai AH, Wani Z (2015). Comparative proteomics and global genome-wide expression data implicate role of ARMC8 in lung cancer. Asian Pac J Cancer Prev.

[25] Fan S, Xu Y, Li X, Tie L, Pan Y, Li X (2014). Opposite angiogenic outcome of curcumin against ischemia and Lewis lung cancer models: in silico, in vitro and in vivo studies. Biochim Biophys Acta.

[26] Ayyub A, Saleem M, Fatima I, Tariq A, Hashmi N, Musharraf SG (2016). Glycosylated Alpha-1-acid glycoprotein 1 as a potential lung cancer serum biomarker. Int J Biochem Cell Biol.

[27] Flores-Perez A, Marchat LA, Sanchez LL, Romero-Zamora D, Arechaga-Ocampo E, Ramirez-Torres N (2016). Differential proteomic analysis reveals that EGCG inhibits HDGF and activates apoptosis to increase the sensitivity of non-small cells lung cancer to chemotherapy. Proteomic Clin Appl.

[28] Giusti L, Iacconi P, Da Valle Y, Ciregia F, Ventroni T, Donadio E (2012). A proteomic profile of washing fluid from the colorectal tract to search for potential biomarkers of colon cancer. Mol Biosyst.

[29] Baek JY, Yeo HY, Chang HJ, Kim KH, Kim SY, Park JW (2014). Serpin B5 is a CEA-interacting biomarker for colorectal cancer. Int J Cancer.

[30] Liu W, Ma Y, Huang L, Peng J, Zhang P, Zhang H (2010). Identification of HSP27 as a potential tumor marker for colorectal cancer by the two-dimensional polyacrylamide gel electrophoresis. Mol Biol Rep.

[31] Vieira de Ribeiro AJ, Sandim V, Ornellas AA, Reis RS, Domont G, Alves G (2013). Differencial proteome of clear-cell renal cell carcinoma (ccRCC) tissues. Int Braz J Urol.

[32] Vasudev NS, Ferguson RE, Cairns DA, Stanley AJ, Selby PJ, Banks RE (2008). Serum biomarker discovery in renal cancer using 2-DE and prefractionation by immunodepletion and isoelectric focusing; increasing coverage or more of the same?. Proteomics.

[33] Kim DS, Choi YP, Kang S, Gao MQ, Kim B, Park HR (2010). Panel of candidate biomarkers for renal cell carcinoma. J Proteome Res.

[34] Sandim V, Pereira Dde A, Kalume DE, Oliveira-Carvalho AL, Ornellas AA, Soares MR (2016). Proteomic analysis reveals differentially secreted proteins in the urine from patients with clear cell renal cell carcinoma. Urol Oncol.

[35] Li M, Yin J, Mao N, Pan L (2013). Upregulation of phosphorylated cofilin 1 correlates with taxol resistance in human ovarian cancer in vitro and in vivo. Oncol Rep.

[36] Ahmed N, Barker G, Oliva KT, Hoffmann P, Riley C, Reeve S (2004). Proteomic-based identification of haptoglobin-1 precursor as a novel circulating biomarker of ovarian cancer. Br J Cancer.

[37] Liu XX, Ye H, Wang P, Li LX, Zhang Y, Zhang JY (2017). Proteomic-based identification of HSP70 as a tumor-associated antigen in ovarian cancer. Oncol Rep.

[38] Lorkova L, Pospisilova J, Lacheta J, Leahomschi S, Zivny J, Cibula D (2012). Decreased concentrations of retinol-binding protein 4 in sera of epithelial ovarian cancer patients: a potential biomarker identified by proteomics. Oncol Rep.

[39] Di Michele M, Marcone S, Cicchillitti L, Della Corte A, Ferlini C, Scambia G (2010). Glycoproteomics of paclitaxel resistance in human epithelial ovarian cancer cell lines: towards the identification of putative biomarkers. J Proteomics.

[40] Dehghan-Nayeri N, Eshghi P, Pour KG, Rezaei-Tavirani M, Omrani MD, Gharehbaghian A (2017). Differential expression pattern of protein markers for predicting chemosensitivity of dexamethasone-based chemotherapy of B cell acute lymphoblastic leukemia. Cancer Chemother Pharmacol.

[41] Tripathi S, Kumar YB, Agrawal A, Prabhakar A, Joshi SS (2016). Microdevice for plasma separation from whole human blood using bio-physical and geometrical effects. Sci Rep.

[42] Lim LC, Looi ML, Zakaria SZS, Sagap I, Rose IM, Chin SF (2016). Identification of differentially expressed proteins in the serum of colorectal cancer patients using 2D-DIGE proteomics analysis. Pathol Oncol Res.

[43] Kuhajda FP, Piantadosi S, Pasternack GR (1989). Haptoglobin-related protein (Hpr) epitopes in breast cancer as a predictor of recurrence of the disease. New Engl J Med.

[44] Wang Q, Yu Q, Lin Q, Duan Y (2015). Emerging salivary biomarkers by mass spectrometry. Clin Chim Acta.

[45] Huang da W, Sherman BT, Lempicki RA (2009). Systematic and integrative analysis of large gene lists using DAVID bioinformatics resources. Nat Protoc.

[46] Stelzer G, Rosen N, Plaschkes I, Zimmerman S, Twik M, Fishilevich S (2016). The GeneCards Suite: From Gene Data Mining to Disease Genome Sequence Analyses. Curr Protoc Bioinformatic.

[47] Bresalier RS, Byrd JC, Tessler D, Lebel J, Koomen J, Hawke D (2004). A circulating ligand for galectin-3 is a haptoglobin-related glycoprotein elevated in individuals with colon cancer. Gastroenterology.

[48] Li J, Tian W, Liu X, Hu S, Zhang B, Li X (2013). [Screening and mass spectrometry analysis of differentially expressed proteins of plasm between laryngocarcinoma and healthy individuals]. J Clin Otorhinolaryngol.

[49] Ghuman S, Van Hemelrijck M, Garmo H, Holmberg L, Malmstrom H, Lambe M (2017). Serum inflammatory markers and colorectal cancer risk and survival. Br J Cancer.

[50] Sun L, Hu S, Yu L, Guo C, Sun L, Yang Z (2016). Serum haptoglobin as a novel molecular biomarker predicting colorectal cancer hepatic metastasis. Int J Cancer.

[51] Sun L, Pan J, Peng L, Fang L, Zhao X, Sun L (2012). Combination of haptoglobin and osteopontin could predict colorectal cancer hepatic metastasis. Ann Surg Oncol.

[52] Dowling P, Clarke C, Hennessy K, Torralbo-Lopez B, Ballot J, Crown J (2012). Analysis of acute-phase proteins, AHSG, C3, CLI, HP and SAA, reveals distinctive expression patterns associated with breast, colorectal and lung cancer. Int J Cancer.

[53] Milano G, Cooper EH, Goligher JC, Giles GR, Neville AM (1978). Serum prealbumin, retinol-binding protein, transferrin, and albumin levels in patients with large bowel cancer. J Natl Cancer Inst.

[54] Shibutani M, Maeda K, Nagahara H, Iseki Y, Ikeya T, Hirakawa K (2016). Prognostic significance of the preoperative ratio of C-reactive protein to albumin in patients with colorectal cancer. Anticancer Res.

[55] Boonpipattanapong T, Chewatanakornkul S (2006). Preoperative carcinoembryonic antigen and albumin in predicting survival in patients with colon and rectal carcinomas. J Clin Gastroenterol.

[56] Dixon MR, Haukoos JS, Udani SM, Naghi JJ, Arnell TD, Kumar RR (2003). Carcinoembryonic antigen and albumin predict survival in patients with advanced colon and rectal cancer. Arch Surg.

[57] Liu Y, Chen S, Zheng C, Ding M, Zhang L, Wang L (2017). The prognostic value of the preoperative c-reactive protein/albumin ratio in ovarian cancer. BMC Cancer.

[58] Kakazu E, Kondo Y, Kogure T, Ninomiya M, Kimura O, Iwata T (2013). Supplementation of branched-chain amino acids maintains the serum albumin level in the course of hepatocellular carcinoma recurrence. Tohoku J Exp Med.

[59] Soudy R, Etayash H, Bahadorani K, Lavasanifar A, Kaur K (2017). Breast cancer targeting peptide binds keratin 1: a new molecular marker for targeted drug delivery to breast cancer. Mol Pharm.

[60] Blanckaert V, Kerviel V, Lepinay A, Joubert-Durigneux V, Hondermarck H, Chenais B (2015). Docosahexaenoic acid inhibits the invasion of MDA-MB-231 breast cancer cells through upregulation of cytokeratin-1. Int J Oncol.

[61] Tang S, Huang W, Zhong M, Yin L, Jiang H, Hou S (2012). Identification Keratin 1 as a cDDP-resistant protein in nasopharyngeal carcinoma cell lines. J Proteomics.

[62] Wang Y, Wang ZQ, Wang FH, Lei XF, Yan SM, Wang DS (2016). Predictive value of chemotherapy-related high-density lipoprotein cholesterol (HDL) elevation in patients with colorectal cancer receiving adjuvant chemotherapy: an exploratory analysis of 851 cases. Oncotarget.

[63] Chandler PD, Song Y, Lin J, Zhang S, Sesso HD, Mora S (2016). Lipid biomarkers and long-term risk of cancer in the Women's Health Study. Am J Clin Nutr.

[64] Borgquist S, Butt T, Almgren P, Shiffman D, Stocks T, Orho-Melander M (2016). Apolipoproteins, lipids and risk of cancer. Int J Cancer.

[65] Cine N, Baykal AT, Sunnetci D, Canturk Z, Serhatli M, Savli H (2014). Identification of ApoA1, HPX and POTEE genes by omic analysis in breast cancer. Oncol Rep.

[66] Liu X, Zheng W, Wang W, Shen H, Liu L, Lou W (2017). A new panel of pancreatic cancer biomarkers discovered using a mass spectrometry-based pipeline. Br J Cancer.

[67] Zhao J, Cheng W, He X, Liu Y, Li J, Sun J (2018). Construction of a specific SVM classifier and identification of molecular markers for lung adenocarcinoma based on lncRNA-miRNA-mRNA network. Onco Targets Ther.

[68] Zhang C, Leng W, Sun C, Lu T, Chen Z, Men X (2018). Urine proteome profiling predicts lung cancer from control cases and other tumors. EBioMedicine.

[69] Repetto O, Maiero S, Magris R, Miolo G, Cozzi MR, Steffan A (2018). Quantitative Proteomic Approach Targeted to Fibrinogen beta Chain in Tissue Gastric Carcinoma. Int J Mol Sci.

[70] Linden M, Segersten U, Runeson M, Wester K, Busch C, Pettersson U (2013). Tumour expression of bladder cancer-associated urinary proteins. BJU Int.

[71] Wang Y, Shan Q, Hou G, Zhang J, Bai J, Lv X (2016). Discovery of potential colorectal cancer serum biomarkers through quantitative proteomics on the colonic tissue interstitial fluids from the AOM-DSS mouse model. J Proteomics.

[72] Hoffmann K, Firth MJ, Beesley AH, Freitas JR, Ford J, Senanayake S (2008). Prediction of relapse in paediatric pre-B acute lymphoblastic leukaemia using a three-gene risk index. Br J Hematol.

[73] Zhi H, Zhang J, Hu G, Lu J, Wang X, Zhou C (2003). The deregulation of arachidonic acid metabolism‐related genes in human esophageal squamous cell carcinoma. Int J Cancer.

[74] Ma XJ, Dahiya S, Richardson E, Erlander M, Sgroi DC (2009). Gene expression profiling of the tumor microenvironment during breast cancer progression. Breast Cancer Res.

[75] Zhu D, Zhong Y, Wu H, Ye L, Wang J, Li Y (2013). Predicting metachronous liver metastasis from colorectal cancer using serum proteomic fingerprinting. J Surg Res.

[76] Kim H, Kim K, Yu SJ, Jang ES, Yu J, Cho G (2013). Development of biomarkers for screening hepatocellular carcinoma using global data mining and multiple reaction monitoring. PloS One.

[77] Ornellas P, Ornellas AA, Chinello C, Gianazza E, Mainini V, Cazzaniga M (2012). Downregulation of C3 and C4A/B complement factor fragments in plasma from patients with squamous cell carcinoma of the penis. Int Braz J Urol.

[78] Gao F, Zhang X, Whang S, Zheng C (2014). Prognostic impact of plasma ORM2 levels in patients with stage II colorectal cancer. Ann Clin Lab Sci.

[79] Zhang X, Xiao Z, Liu X, Du L, Wang L, Wang S (2012). The potential role of ORM2 in the development of colorectal cancer. PloS One.

[80] Rucksaken R, Charoensuk L, Pinlaor P, Pairojkul C, Khuntikeo N, Pinlaor S (2017). Plasma orosomucoid 2 as a potential risk marker of cholangiocarcinoma. Cancer Biomarker.

[81] Fang T, Cui M, Sun J, Ge C, Zhao F, Zhang L (2015). Orosomucoid 2 inhibits tumor metastasis and is upregulated by CCAAT/enhancer binding protein β in hepatocellular carcinomas. Oncotarget.

[82] Rucksaken R, Khoontawad J, Roytrakul S, Pinlaor P, Hiraku Y, Wongkham C (2013). Proteomic analysis to identify plasma orosomucoid 2 and kinesin 18A as potential biomarkers of cholangiocarcinoma. Cancer Biomarker.

[83] Duché JC, Urien S, Simon N, Malaurie E, Monnet I, Barré J (2000). Expression of the genetic variants of human alpha-1-acid glycoprotein in cancer. Clin Biochem.

[84] Siegerink B, Rosendaal FR, Algra A (2009). Genetic variation in fibrinogen; its relationship to fibrinogen levels and the risk of myocardial infarction and ischemic stroke. J Thromb Haemost.

[85] Vu D, Bolton-Maggs PH, Parr JR, Morris MA, de Moerloose P, Neerman-Arbez M (2003). Congenital afibrinogenemia: identification and expression of a missense mutation in FGB impairing fibrinogen secretion. Blood.

[86] Wojtukiewicz MZ, Zacharski LR, Memoli VA, Kisiel W, Kudryk BJ, Rousseau SM (1989). Indirect activation of blood coagulation in colon cancer. Thromb Haemost.

[87] Tan Z, Zhang M, Han Q, Wen J, Luo K, Lin P (2017). A novel blood tool of cancer prognosis in esophageal squamous cell carcinoma: the Fibrinogen/Albumin Ratio. J Cancer.

[88] Lin Y, Liu Z, Qiu Y, Zhang J, Wu H, Liang R (2018). Clinical significance of plasma D-dimer and fibrinogen in digestive cancer: A systematic review and meta-analysis. Eur J Surg Oncol.

[89] Troppan KT, Melchardt T, Wenzl K, Schlick K, Deutsch A, Bullock MD (2016). The clinical significance of fibrinogen plasma levels in patients with diffuse large B cell lymphoma. J Clin Pathol.

[90] Lu ZL, Chen YJ, Jing XY, Wang NN, Zhang T, Hu CJ (2018). Detection and Identification of Serum Peptides Biomarker in Papillary Thyroid Cancer. Int Med J Exp Clin Res.

[91] Kolodziejczyk J, Ponczek MB (2013). The role of fibrinogen, fibrin and fibrin(ogen) degradation products (FDPs) in tumor progression. Contemp Oncol (Pozn).

[92] Davalos D, Akassoglou K (2012). Fibrinogen as a key regulator of inflammation in disease. Semin Immunopathol.

[93] Mitter C, Zielinski C (1991). Plasma levels of D-dimer: a crosslinked fibrin-degradation product in female breast cancer. J Cancer Res Clin Oncol.

[94] Repetto O, Maiero S, Magris R, Miolo G, Cozzi M, Steffan A (2018). Quantitative proteomic approach targeted to fibrinogen β chain in tissue gastric carcinoma. Int J Mol Sci.

[95] Schäfer M, Werner S (2008). Cancer as an overhealing wound: an old hypothesis revisited. Nat Rev Mol Cell Biol.

[96] Falus A, Kramer J, Walcz E, Varga Z, Setalo J, Jobst K (1989). Unequal expression of complement C4A and C4B genes in rheumatoid synovial cells, human monocytoid and hepatoma-derived cell lines. Immunology.

[97] Afshar-Kharghan V (2017). The role of the complement system in cancer. J Clin Invest.

[98] Pio R (2006). Control of complement activation by cancer cells and its implications in antibody-mediated cancer immunotherapy. Inmunology.

[99] Pio R, Corrales L, Lambris JD (2014). The role of complement in tumor growth. Adv Exp Med Biol.

[100] Taneja S, Ahmad I, Sen S, Kumar S, Arora R, Gupta VK (2011). Plasma peptidome profiling of acute hepatitis E patients by MALDI-TOF/TOF. Proteome Sci.

